# Optimized microwave extraction, characterization and antioxidant capacity of biological polysaccharides from *Eucommia ulmoides* Oliver leaf

**DOI:** 10.1038/s41598-018-24957-0

**Published:** 2018-04-26

**Authors:** Jikun Xu, Huijie Hou, Jingping Hu, Bingchuan Liu

**Affiliations:** School of Environmental Science & Engineering, Huazhong University of Science and Technology, Wuhan, 430074 China

## Abstract

Microwave-induced technique was combined with response surface methodology for optimizing the isolation of polysaccharides from *Eucommia ulmoides* Oliver leaf. The maximum polysaccharides yield of 12.31% was achieved by microwave extraction at 74 °C for 15 min with a solid to liquid ratio of 1:29 g/mL, which agreed with the predicted value and was 2.9-fold higher than that of the conventional heat-reflux extraction method. The dominant bioactive constituent in extracts was chlorogenic acid (1.3–1.9%), followed by geniposidic acid (1.0–1.7%). The polysaccharides from the optimized extraction had a high molecular weight and polydispersity (*M*_w_ 38,830 g/mol, *M*_w_/*M*_n_ 2.19), as compared to the fraction prepared in the absence of microwave (*M*_w_ 12,055 g/mol, *M*_w_/*M*_n_ 1.26). Glucose was the dominant sugar component (38.2–39.1%) of heterogeneous polysaccharides which belonged to a structure of β-type acidic heteropolysaccharides with a glucan group and highly branched degree. The polysaccharides showed a higher DPPH radical scavenging index (0.87–1.22) than BHT (0.41) but lower than BHA (3.56), which can act as a favorable antioxidant in functional food.

## Introduction

Because of the potent nontoxic effects and therapeutic properties, the research on the biological polysaccharides and associated antioxidants from plant resources has attracted an increasing interest in the last decades with a diversified range of applications. The water-soluble polysaccharides and water extracts from Chinese traditional herb materials exhibited efficient bioactivities, including antioxidant, immunomodulation and anti-tumor, as reported by the previous studies^1–4^. These abundant polysaccharides and bioactive components are ideal candidates for potential utilization in food and biomedical industries. *Eucommia ulmoides* Oliver (*E*. *ulmoides*), also called Du-Zhong in Chinese, which belongs to the Eucommiaceae family, is one of the most famous traditional woody plants growing in China for nutrition and folk medicine. Used in the treatment of hypercholesterolemia, hypertension, and fatty liver diseases, Du-zhong tea (refers to the aqueous extract of *E*. *ulmoides* leaf) has been commonly adopted and already known as a functional health food^5–8^. In addition, the existence of phenolic composition results in various beneficial health properties of *E*. *ulmoides*, which occurs in nature as a mixture of esters, ethers, or free acids^9^. It has been found that the bioactive constituents are rich in *E*. *ulmoides* leaf, such as polyphenolic acids, flavonoids, iridoids, and nutrients^10,11^. The main effective constituents have been demonstrated to extend the mean lifespan and protect against stress-provoked influenza viral infection^10–12^. Polysaccharides are a widespread biological macromolecule composed of the same or different monosaccharides and uronic acids that condensed by glycosidic linkages, and are essential ingredients for all living organisms^13^. Polysaccharides are widely found in plants, microorganisms, bacteria, fungi and seaweed, and they are closely related to many physiological behaviors of life^3^. Moreover, polysaccharides in both simple and complex glycoconjugated form have the potential to support a variety of functions, such as antioxidant, antivirus, immunomodulation, antidiabetes, hepatoprotection, antitumour, and antifatigue functions^14,15^. It has been found that polysaccharides isolated from *Eucommia ulmoides* leaf possessed an important antioxidant role with effective scavenging activities on radicals, which are suitable for the development of medical reagents^16^.

Therefore, an efficient technique for the extraction of *E*. *ulmoides* leaf polysaccharides (EULP) and antioxidants from *E*. *ulmoides* leaves is imperative. Up to now, hot water technology is the mostly adopted method for the conventional isolation of polysaccharides and active compounds^17^, which is however associated with longer extraction time and lower efficiency. In the effective epuration of products from different plant materials, microwave-assisted extraction (MAE) has been widely conducted due to its accelerated mass transfer between immiscible phases facilitated by the electromagnetic radiation^18,19^. As compared to other methods, response surface methodology (RSM) allows more efficient interpretation and easier arrangement of experiments. RSM is an effective multivariate statistical method for optimizing complex experimental processes. It generates a second-degree polynomial model by regression fitting of response surface analysis to evaluate the polytomy variables and its interaction, and then determine the best level. The most important advantage of RSM is the reduced number of trials on process optimization^20,21^. Box-Behnken design (BBD), a type of RSM, is an independent, spherical, and rotatable quadratic method that consists of three interlocking 2^2^ factorial designs with points locating on the surface of a sphere surrounding the center of the design. It has been successfully exerted to optimize various biochemical and biotechnological processes^22^. Therefore, RSM has been used frequently to optimize the parameters of extraction experiments^23–25^.

In this study, a three-level and three variables BBD was used to model and optimize the extraction parameters (extraction temperature, time, and liquid to solid ratio) of polysaccharides by the MAE process for the elucidation of the different parameters that govern the extraction and structure of polysaccharides from *E*. *ulmoides* leaf. The MAE process optimized by RSM can thus be predicted and controlled for industrial applications. A mathematical model was constructed by RSM to evaluate the effects of the three independent variables and their combinatorial interactions on the extraction yield of target products. A comparative analysis between the MAE and conventional heat-reflux extraction (CHE) methods was also conducted. The eight main types of bioactive compounds associated with *E*. *ulmoides* leaf polysaccharides in the supernatant were identified and quantified by high performance liquid chromatography (HPLC). Analysis of the chemical compositions, structures, and antioxidant capacity of EULP was carried out by ultraviolet-visible spectroscopy (UV-Vis), sugar analysis, gel permeation chromatography (GPC), and Fourier transform infrared spectroscopy (FTIR) for unraveling the possibility of adopting the antioxidant polysaccharides to functional foods.

## Materials and Methods

### Materials and chemicals

Fresh *E*. *ulmoides* leaves were manually harvested from the arboretum of Northwest A & F University, Yangling, China. To remove dusts and impurities deposited on the surface, the leaves were rinsed in water repeatedly and then dried in an oven at 40 °C until constant weight was attained. Subsequently, the dried samples were ground and sieved to obtain fine powder of about 400‒500 μm particle diameter size, and stored in dry and dark environment prior to use.

The standard reagents, such as bioactive components and monosaccharides (National Institutes for Food and Drug Control, Beijing, China), were of chromatographic purity. Other chemical agents, including ethanol, sulfuric acid and phenol, were purchased from Sigma Chemical Company (Beijing, China) and used without further purification.

### Microwave-assisted extraction

The MAE process was conducted to extract the polysaccharides from the leaf samples based on RSM^24^. Briefly, 2.0 g of ground leaf samples were mixed with specific volume (Table 1) of distilled water into a conical flask. The flask was set to a microwave generator (MILESTONE, MicroSYNTH, USA) to perform the extraction under conditions listed in Table 1. The extract was vacuum filtered and the filtrates obtained were evaporated under vacuum at 40 °C to obtain the concentrated filtrates. Then ethanol (95%) with a volume of 3 times of the concentrated filtrate was added slowly to the filtrate with continuous stirring. The precipitate was recovered by centrifugation and lyophilization to obtain polysaccharides EULP-MAE. Then, the supernatant obtained was concentrated and filtered through a 0.22 μm membrane to obtain a clear filtrate prior to quantitative analysis of bioactive compounds. The experimental conditions are listed in Table 1. Polysaccharide fraction EULP-CHE extracted with the CHE method under the conditions optimized for MAE was used as a control sample.

**Table 1:** BBD and the responses for the yields of polysaccharides.

Run	Coded variable levels			Yield of polysaccharides (%)	
	A (ratio)	B (temp.)	C (time)	Experimental	Predicted
1	20	80	10	10.79	10.53
2	20	40	30	8.13	8.39
3	20	60	20	11.07	11.08
4	30	80	20	12.06	12.00
5	20	60	20	11.15	11.08
6	30	60	10	11.22	11.54
7	20	60	20	10.96	11.08
8	20	40	10	8.84	8.46
9	20	80	30	8.96	9.35
10	10	40	30	8.61	8.67
11	10	60	10	8.69	9.01
12	30	40	20	9.45	9.51
13	10	80	20	9.27	9.21
14	10	60	30	9.43	9.11
15	30	60	30	10.53	10.21
16	20	60	20	11.23	11.08
17	20	60	20	10.99	11.08

### Response surface method

On the basis of the results of preliminarily single factor experiments, BBD was used for the optimization using three independent variables^24^. The liquid to solid ratio, extraction temperature, and time were chosen as key parameters and designated as A, B, and C, respectively, as shown in Table 1.

### Analysis of bioactive components

**Figure 1:**
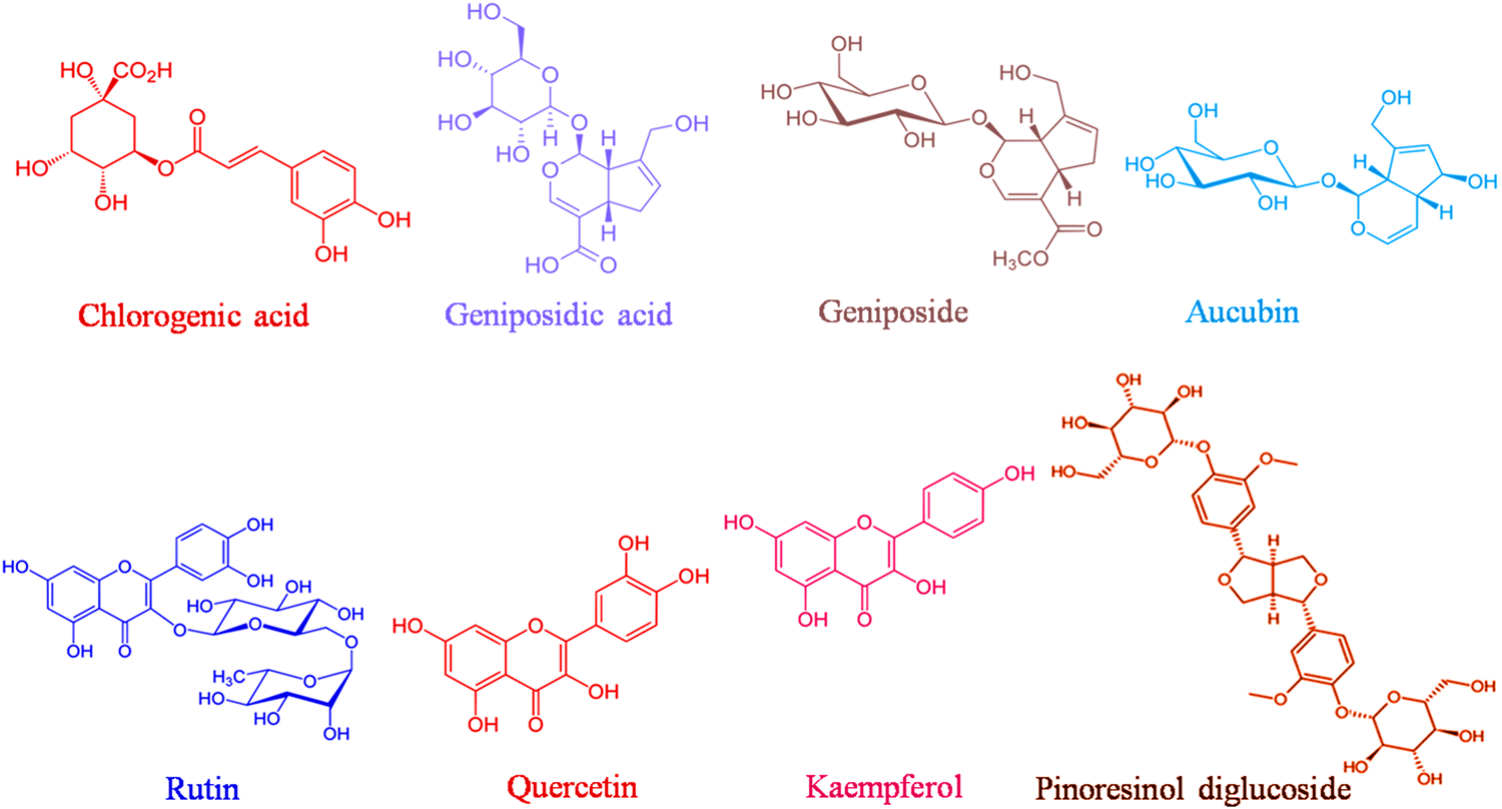
Chemical structures of the major bioactive compounds of *E*. *ulmoides* leaf.

Quantitative analysis of the concentration of chlorogenic acid (CGA), geniposidic acid (GPA), geniposide (GP), rutin (RU), quercetin (QU), kaempferol (KA), aucubin (AU), and pinoresinol diglucoside (PDG) in the extractum from *E*. *ulmoides* leaves were carried out by a HPLC system (Agilent 1200 series, Agilent Technologies, U.S.) following a chromatography analysis procedure reported in the literature^26^. The structural formulas of these bioactive components are illustrated in Fig. 1.

### Structural characterization of polysaccharides

Phenol-sulfuric acid method was adopted for the determination of carbohydrate content of EULP with glucose as the standard reagent following the procedure described by Gan *et al*.^24^. The molecular weights of polysaccharides were determined by GPC following an established procedure^27^. A 2 mg sample of EULP was dissolved in 2 mL mixture of 0.02 M NaCl and 0.005 M sodium phosphate buffer (pH 7.5) prior to measurement. The experiments were performed at least three times. High performance anion exchange chromatography (HPAEC) was employed to determine the monomeric sugars released by the acid hydrolysis of EULP with 1 M H_2_SO_4_ for 2.5 h at 105 °C^27^. FTIR spectra of polysaccharides were conducted using a FTIR Microscope (Thermo Nicolet Corporation, Madison, WI) equipped with a MCT detector cooled by liquid nitrogen^27^.

After adding the mixture of polysaccharide solutions or control (EULP-CHE) in ethanol solution, the DPPH (1,1-diphenyl-2-picrylhydrazyl) radical scavenging effect was measured^28^. The scavenging ability was calculated by the following formula:

$${\rm{Scavenging}}\,{\rm{ability}}\,( \% )=[1-({{\rm{A}}}_{1}-{{\rm{A}}}_{2})/{{\rm{A}}}_{0}]\times 100 \% $$

where A_0_ is the absorbance of water used as the control, A_1_ is the absorbance of the sample, A_2_ is the absorbance of the sample under identical conditions as A_1_ and with water instead of DPPH solution. IC50 was referred as the concentration of EULP with 50% inhibition. Radical scavenging index (RSI) was defined as the count backwards of IC50.

### Statistical analysis

All the data were calculated as the mean of three replicate determinations within significance *p* < 0.05 after analyzing the variance (ANOVA) and processing with SPSS 13.0. The Design Expert software (Stat-Ease, Minneapolis) was utilized for the regression analysis and the optimization of graphics.

## Results and Discussion

### Model fitting and statistical analysis

In the current BBD of RSM, a total of 17 runs were applied in the production of EULP to optimize the three individual parameters. The effects of three MAE conditions, including liquid to solid ratio (A), extraction temperature (B) and time length (C) on the yields of EULP (response values) were investigated (Table 1). As power level alone does not give sufficient information regarding the absorbed microwave energy into the extraction system, *Alfaro* and co-workers introduced a term known as energy density, power per mass for a given unit of time, to investigate the effect of microwave power on MAE^29^. It is reported that once the plant matrix was destroyed by microwave radiation, the active compounds would be released. Higher power level does not give any contribution to the investigation of interaction between microwaves and extraction solvent with the sample. Temperature and microwave power were interrelated, as high microwave power could raise the extraction temperature which has been optimized in this investigation^30^. The process variables, including liquid to solid ratio (10:1–30:1 mL/g), extraction temperature (40–80 °C) and time (10–30 min), were selected by the single parameter analysis. As shown in Table 1, the predicted response (R) of the EULP yield could be calculated by a second-order polynomial equation:

$$\begin{array}{rcl}{\rm{R}}\,( \% ) & = & 11.08+0.91\times {\rm{A}}+0.76\times {\rm{B}}\\  &  & -0.31\times {\rm{C}}+0.49\times {\rm{A}}\times {\rm{B}}-0.36\times {\rm{A}}\times {\rm{C}}-0.28\times \,{\rm{B}}\times {\rm{C}}\\  &  & -0.22\times {{\rm{A}}}^{2}-1.01\times {{\rm{B}}}^{2}-0.89\times {{\rm{C}}}^{2}\end{array}$$

The analysis of variances was employed for selecting the best model matched with the operation results (Table 2). The dominant effect of liquid to solid ratio (A) was highly significant (*F*-value > 50 and *p* < 0.01, Table 2), which implied that the ratio of liquid to solid was interrelated to the EULP yield directly and the most influential factor was the liquid to solid ratio among the variables. Moreover, a significant model was established owing to a high *F* value (18.82) and a low *p*-value (*p* < 0.01) of the equation. The coefficient value (R^2^) of the model was 0.9603, implying a sample variation of 96.03% for the yield of EULP. An excellent correlation between the independent variables was determined by the adjusted correlation coefficient (R^2^_Adj_) of the model.

**Table 2:** Regression coefficient and their significance test of the second-order polynomial model.

Source	Degrees of freedom	Sum of squares	Mean square	*F*-value	*p*-value
Model^a^	9	22.22	2.47	18.82	<0.01
A	1	6.59	6.59	50.21	<0.01
B	1	4.58	4.58	34.87	<0.01
C	1	0.78	0.78	5.91	0.0454
AB	1	0.95	0.95	7.24	0.0310
AC	1	0.51	0.51	3.90	0.0890
BC	1	0.31	0.31	2.69	0.1660
A^2^	1	0.21	0.21	1.59	0.2479
B^2^	1	4.30	4.30	32.73	<0.01
C^2^	1	3.34	3.34	25.42	<0.01

^a^R^2^ = 0.9603, R^2^_Adj_ = 0.9093, C.V. = 3.59%.

### Analysis of response surfaces

**Figure 2:**
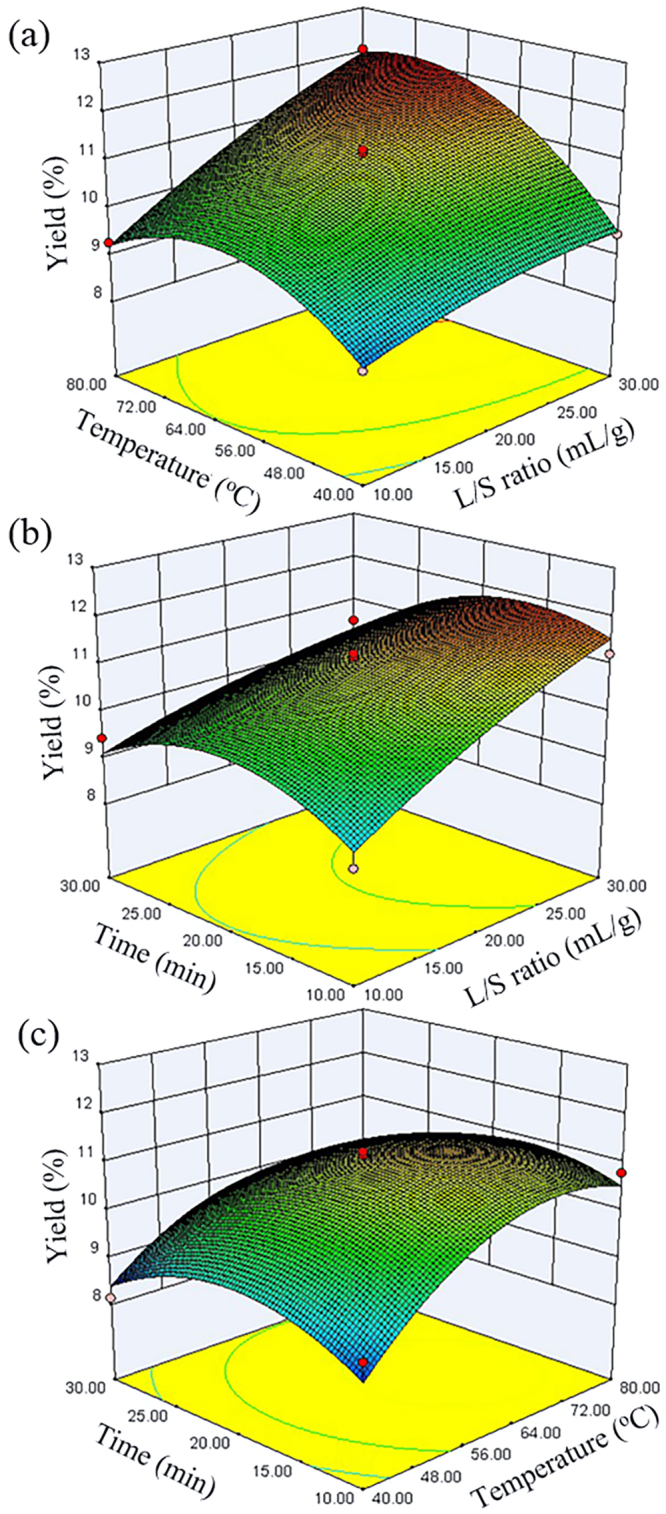
The response surface plots depicting the impact of (**a**) extraction temperature and liquid/solid ratio, (**b**) extraction time and liquid/solid ratio, and (**c**) extraction time and temperature on the yield of EULP.

In each 3D response surface plot, the interaction of two variables was studied simultaneously, while the value of third parameter was set in its middle level, and the results are shown in Fig. 2. It can be noted that both liquid to solid ratio and extraction temperature exerted a quadratic effect on the yield of polysaccharides (Fig. 2a). Moreover, the liquid to solid ratio had a positive linear effect on the EULP yield, and the EULP yield increased as the liquid to solid ratio increased from 10 to 30 during the MAE process. As the temperature was increased from 56 to 72 °C, a significant enhancement in the yield of polysaccharides was achieved (Fig. 2a). However, indiscernible improvement of yield was observed at higher extraction temperatures (72–80 °C), which could be ascribed to the partial degradation of EULP at higher temperature^31^. These findings were in consistent with the fractionation of natural polysaccharides from *Zizyphus jujuba* cv. *Jinsixiaozao*^21^ and *Anastatica hierochuntica*^32^. The effects of liquid to solid ratio (A) and extraction time (C), and their reciprocal correlations on EULP yield at 60 °C were depicted in Fig. 2b. The extraction yield increased initially with an increment of the liquid to solid ratio and extraction time. The longer extraction time led to a slight increase in EULP yield whereas the increase of liquid to solid ratio induced an exponential increase of EULP yield during the first 20 min, and the improvement of yield gradually became constant from 20 to 30 min. The results indicated that the EULP yield ranged from 8.69% to 11.23% at 60 °C (Fig. 2b) and the correlations between the variable parameters were insignificant (*p* > 0.05, Table 2). The effects of extraction temperature (B) and time (C) on the EULP yield are presented in Fig. 2c. When the value of A was designed at 1:20, the maximal EULP yield of 11.23% was achieved at an extraction temperature of 60 °C and a processing time of 20 min.

### Optimization of extraction parameters and validation of the model

Under the optimized experimental conditions for the MAE process, the theoretical maximum EULP yield of 12.35%, corresponding to initial weight of dried *E*. *ulmoides* leaf, was acquired at 73.65 °C for 15.16 min with a liquid to solid ratio of 28.9. A verified experiment was conducted under the optimal conditions to confirm the accuracy of the model. To validate the actual operation conditions, the EULP yield of 12.31% was achieved at 74 °C for 15 min with a liquid to solid ratio of 29, which matched the predicted yield of the RSM model. These results confirmed that the response model is sufficient to reflect the good correlation between the theoretical optimization and experimental value.

As a control sample, EULP-CHE was extracted with the CHE method at the optimized conditions (the liquid to *E*. *ulmoides* leaf ratio of 29 mL/g, extraction at 74 °C in water bath for 15 min), and a yield of 5.62% was achieved. The comparison between the CHE and MAE methods indicated that microwave irradiation can greatly improve the extraction efficiency (12.31% *vs*. 5.62%) and the purity of polysaccharides (89.5% *vs*. 84.2%). This result was consistent with the previous studies of polysaccharides extraction^33–36^. The high extraction efficiency of EULP by the MAE process can be attributed to the breaking of the cells from *E*. *ulmoides* leaves by the microwave treatment, which facilitates the dissolution of the polysaccharide fractions in the solvent^33^. The result demonstrated that microwave-assisted extraction of polysaccharides from *E*. *ulmoides* leaf was a time-saving, efficient and high yield method.

### Contents of antioxidants

**Figure 3:**
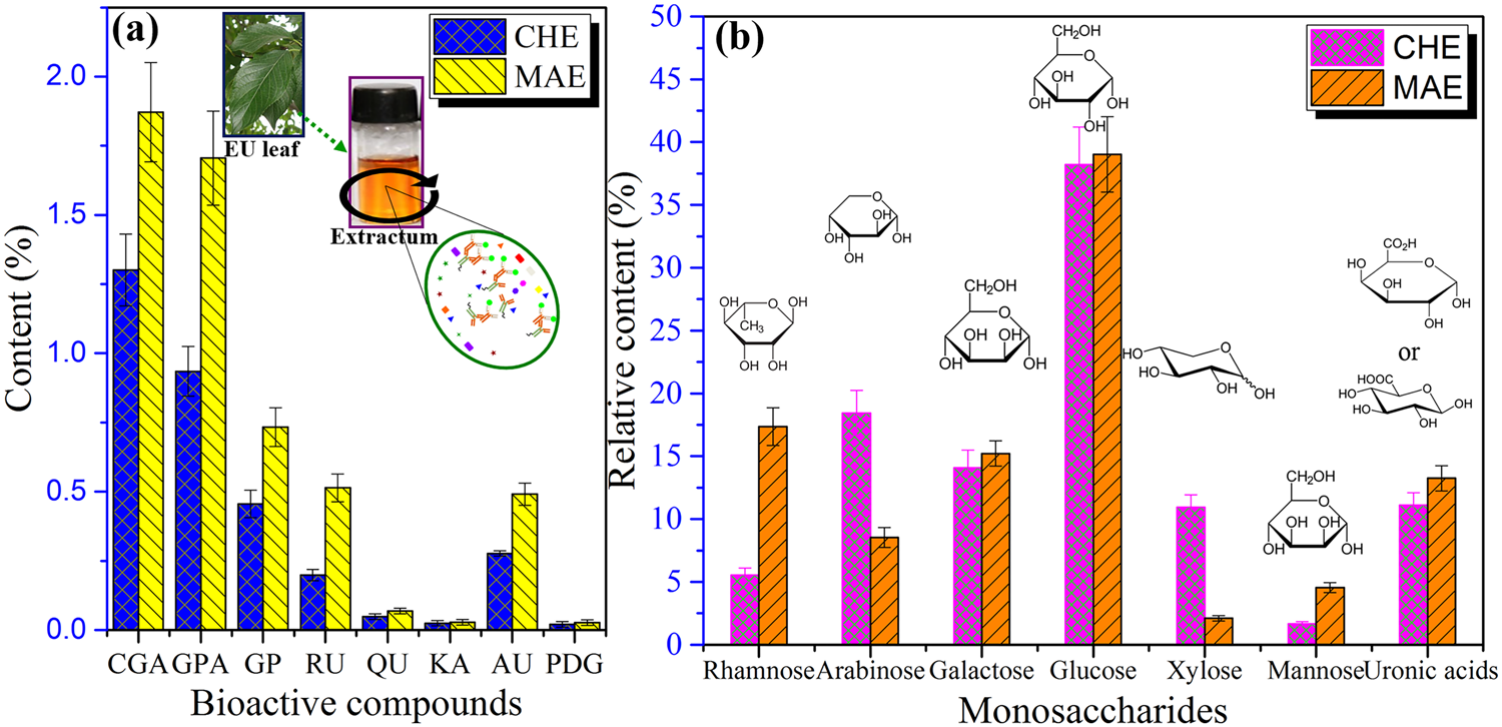
(**a**) The amounts of bioactive components in aqueous *E*. *ulmoides* leaf extracts after conventional heat reflux extraction (CHE) and microwave assisted extraction (MAE). CGA, chlorogenic acid; GPA, geniposidic acid; GP, geniposide; RU, rutin; QU, quercetin; KA kaempferol; AU, aucubin; PDG, pinoresinol diglucoside. (**b**) The chemical compositions of polysaccharides (relative percent of dry samples, w/w). CHE, conventional heat reflux extraction method; MAE, microwave assisted extraction method.

Phenolics are the most effective antioxidants derived from plants^37,38^. In the present study, the phenolics were simultaneously extracted from *E*. *ulmoides* leaf together with polysaccharides. The existence of water soluble polysaccharides-polyphenolic conjugates could make a contribution to the antioxidant activity^39^. Therefore, the extraction and determination of phenolics are the main steps to obtain bioactive ingredients associated with polysaccharides from medicinal plant materials. As the fractionation and solubilization of some polysaccharides occur along aqueous extraction, the liquid phase is expected to have an increased phenolic content and improved antioxidant activity. The contents of CGA, GPA, GP, RU, QU, KA, AU, and PDG from *E*. *ulmoides* leaves were investigated and the results are shown in Fig. 3a. As can be seen, the contents of natural antioxidants were significantly affected by the heating mode in the extraction process. The MAE exhibited significant advantages in terms of high extraction efficiency of bioactive components from the plant materials as compared to the CHE. The total content of eight main bioactive components extracted by the MAE method (5.45%) was apparently higher than that extracted by the CHE method (3.26%). The result indicated that the MAE processing can greatly accelerate the release of natural antioxidants. This result was mainly ascribed to the more efficient transmission of microwave irradiation to *E*. *ulmoides* leaf through molecular interaction with the electromagnetic field and the mechanical effects of microjetting and microstreaming, thus facilitated a rapid dissolution of EULP into solvent^40^. The major free active compound in water extracts was CGA, followed by GPA, GP, RU, and AU, together with minor amounts of QA, KA, and PDG. The antioxidant activity of extracts have been reported in the literature to present a marked correlation with the levels of soluble phenolic content^26^. Apparently, CGA was the dominant antioxidant in both extracts using the CHE and MAE methods, amounting to 1.30% and 1.87%, respectively. In addition, GPA appeared to be the secondary major antioxidant, comprising 0.94% and 1.71%, respectively. Other bioactive components were observed as noticeable amounts. Moreover, the contents of CGA, GPA, GP, RU, and AU in the sample collected by the MAE process were slightly higher as compared to those by the CHE method. However, the fluctuations of the yields of QU, KA and PDG were small. This indicated that the heating mode significantly influenced the content of each functional constituent to different extents, which may result from the differences in their chemical structures and metabolism process. In summary, the MAE is favorable as compared to the CHE method for screening effective components.

### Structural characterization of polysaccharides

#### Compositional analysis of EULP

From the sugar analysis of the extracted polysaccharides (Fig. 3b), the polysaccharide fractions contained relatively high levels of carbohydrates, ranging from 84.2% (EULP-CHE) to 89.5% (EULP-MAE), indicating that the purity of EULP-MAE was higher than that of EULP-CHE. Based on UV-spectral data, the remaining mass was identified predominantly as proteins which may have been associated with these polysaccharides.

The monosaccharide composition of polysaccharides after acid hydrolysis was identified and determined through sugar analysis^27^. Usually, the treatment with acid and high temperature leads to the partial degradation of carbohydrates to form byproducts. Among them, the monosaccharides originated from polysaccharides could be degraded into several byproducts, such as furfural, 5-hydroxymethylfurfural and carboxylic acids. Obviously, glucose was a dominant sugar component in the EULP-MAE and EULP-CHE samples, comprising 38.2–39.1% of the total sugar composition, whereas arabinose (8.5–18.5%), rhamnose (5.6–17.4%), and galactose (14.1–15.2%) were present in small amounts. Xylose (2.1–10.9%) and mannose (1.7–4.5%) were observed as the minor constituents in all polysaccharides. In addition, it was found that a certain amount of uronic acid (11.1–13.3%) was presented. The first polysaccharide, designated EULP-CHE, contained L-rhamnose, L-arabinose, D-galactose, D-glucose, D-xylose, D-mannose, D-glucuronic acid, and D-galacturonic acid, in the approximate molar ratios of 9:32:20:55:19:2:14:1. The other (EULP-MAE) contained the alike monosaccharides in the approximate molar ratios of 7:4:6:14:1:2:3:1. Interestingly, it was found that the discrepancy between the contents of rhamnose, arabinose, and xylose in the two polysaccharides were significant, suggesting the differences of sugar composition in polysaccharides were ascribed to the different extraction processes. The possible interpretation was that the linkages, such as the hydrogen bonds, between the polysaccharides and the other components in the plant cell walls of *E*. *ulmoides* leaf were partially cleaved, which led to different contents of sugars. It is worth mentioning that both EULP-CHE and EULP-MAE contained a major fraction of glucose, amounting to 38.2% and 39.1%, respectively. This indicated that some β-glucans or starch were probably presented in the isolated polysaccharides and the microwave-assisted treatments may favor the release of β-glucans.

#### Molecular weight distribution

**Figure 4:**
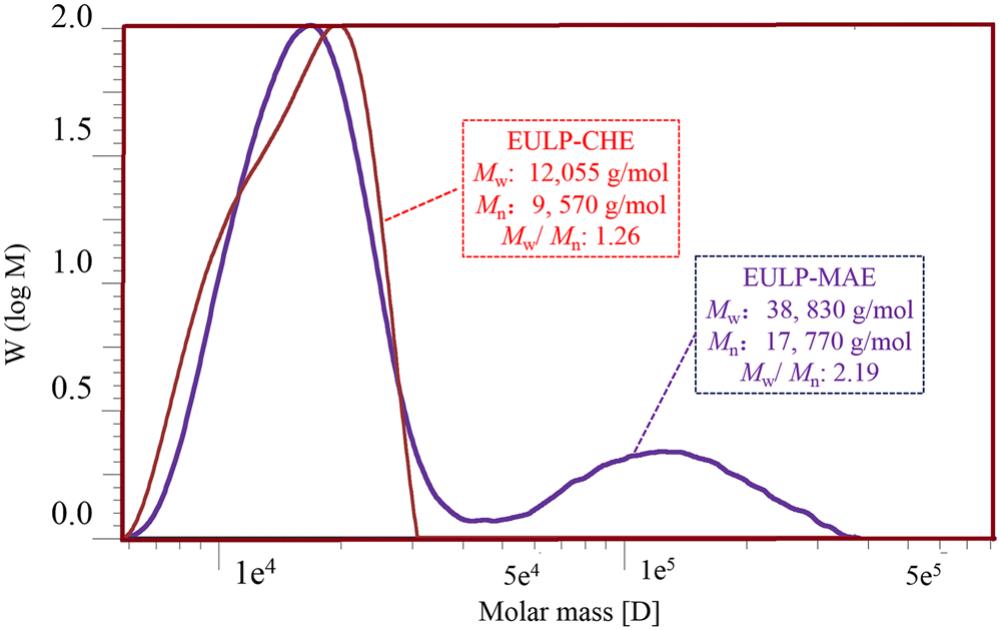
Molecular weight distribution curves of polysaccharides. EULP-CHE, *E*. *ulmoides* leaf polysaccharides via conventional heat reflux extraction; EULP-MAE, *E*. *ulmoides* leaf polysaccharides via microwave assisted extraction.

The weight-average (*M*_w_) and number-average (*M*_n_) molecular weights, and polydispersity (*M*_w_/*M*_n_) of the polysaccharide fractions were determined to estimate the effects of the microwave heating on the molecular structures. The molecular weight distribution of fractions was analyzed by GPC and the representative spectra are presented in Fig. 4. As can be seen from this figure, when the MAE was conducted at the optimal conditions, the polysaccharides had a high molecular weight and polydispersity (*M*_w_ 38,830 g/mol, *M*_w_/*M*_n_ 2.19). From molecular weight analysis of the polysaccharides EULP-CHE, a narrower distribution and apparent reduction of the molecular weight were observed (*M*_w_ 12,055 g/mol, *M*_w_/*M*_n_ 1.26). These discrepancies in molecular weight were probably due to the nature of the sample and the various isolation conditions used, in which the backbone chain and substituent of polysaccharides may play a very important role in manipulating the molecular weight. Furthermore, molecular weight distribution curves also illustrated the structural feature of the polysaccharides obtained by different extraction processes. It was found that EULP-MAE exhibited bimodal molecular weight distribution. This suggested that the microwave had considerable linkage-breaking effects, which ultimately led to heterogeneous molecules, as confirmed by polydispersity. However, the polysaccharide fraction EULP-CHE displayed unimodal molecular weight distribution curves. This phenomenon could be ascribed to slow mass transfer between the solvent and *E*. *ulmoides* leaf when performed with the CHE. This work proved that the MAE method is a feasible way to explore the potential application of polysaccharides-based bioactive components in the food industry.

#### FTIR analysis

**Figure 5:**
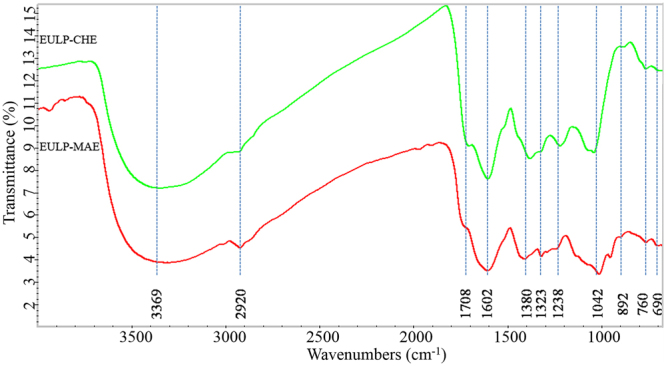
FTIR spectra of the polysaccharide fractions from *E*. *ulmoides* leaf using conventional heat reflux extraction (EULP-CHE) and microwave assisted extraction (EULP-MAE) methods.

To further investigate the structural heterogeneity between the bioactive hydrophilic polysaccharides, FTIR spectra of EULP-CHE and EULP-MAE were recorded (Fig. 5). The spectra of the fractions show minor changes in the peak intensities, indicating that these water-extractable polysaccharides had an analogous chemical structure. The FTIR spectra show an intense and broad band at 3369 cm^−1^ representing hydroxyl groups^41^. The adsorption of anti-symmetrical stretching vibration in C–H group is located at 2926 cm^−1^, the bands around 1745 cm^−1^ suggest the presence of ester carbonyl groups (C=O) of uronic acids in polysaccharides, and those in the region of 1652 cm^−1^ are due to bound water^41,42^. A weak symmetrical stretching peak at approximately 1400–1200 cm^−1^ can be attributed to the presence of carboxyl groups^41^. The peaks in the range 1200–1000 cm^−1^ corresponded to the ring vibrations overlapping with stretching vibrations of the (C–*O*–C) glycosidic linkage vibration and (C–OH) side groups^43^. As can be seen in Fig. 5, a notable shift of this peak appeared at 1042 cm^–1^, which corresponded to the C–C and C–O stretching modes and the glycosidic band (C–*O*–C) vibration in polysaccharides^44^. This finding implied that a typical absorbance for xylans was presented in the polysaccharide fractions. The weak absorbance at 1238 cm^−1^ was due to the fraction EULP-CHE, and some acetyl groups attached to the polysaccharides were confirmed by the weak peak at 1238 cm^−1^. This indicated that more acetyl groups linked to the polysaccharides were degraded in the presence of microwave irradiation, thus only a portion of the acetyl groups were preserved in EULP-MAE. The absorption at 913 cm^−1^ was related to the β-pyranose of glucose. Another absorbance at 892 cm^−1^ was related to the C1 group frequency or ring frequency in the polysaccharides, which corresponded to the β-glycosidic bonds between the D-mannose units. Moreover, the band at 760 cm^−1^ showed a β-configuration of the sugar units. Based on the FTIR and sugar analysis of EULP-CHE and EULP-MAE, it can be assumed that these polymers belonged to a structure of β-type acidic heteropolysaccharides with a glucan group and highly branched degree.

#### Antioxidant capacity of EULP *in vitro*

**Figure 6:**
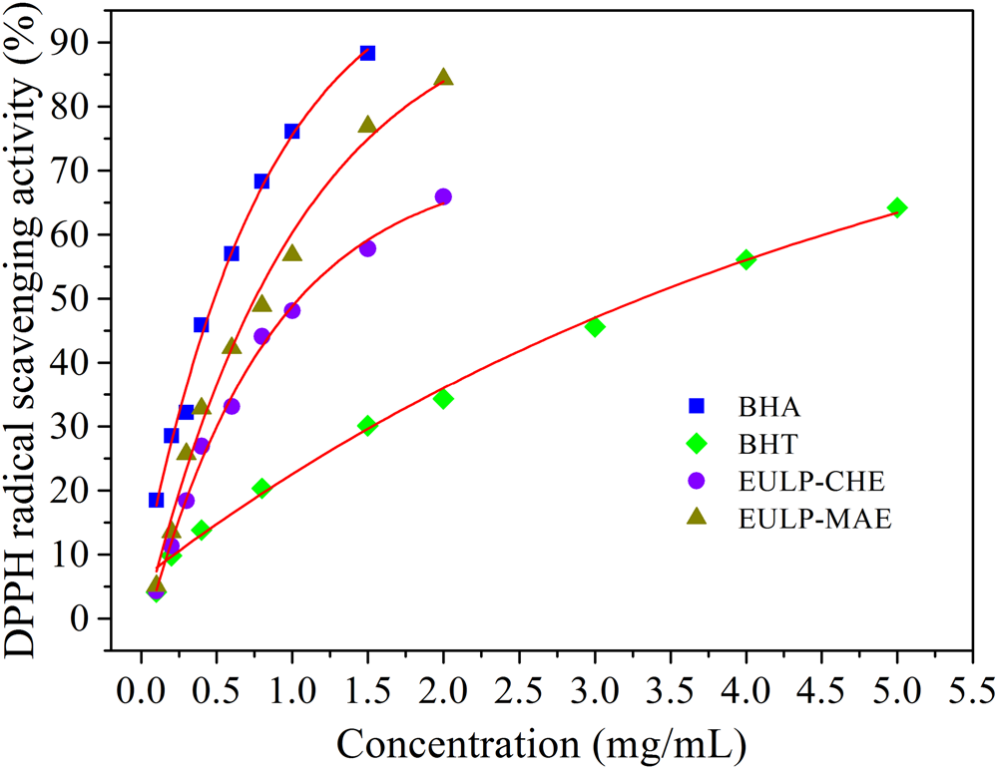
Scavenging effect of EULP-CHE and EULP-MAE on DPPH radical.

As compared to the classic commercial antioxidants, including BHA and BHT, the antioxidant activity of EULP against DPPH radical is presented in Fig. 6. As shown in this figure, the two polysaccharides exhibited a noticeable and dose-dependent DPPH radical scavenging activity, which was ascribed to the hydrogen donating capacity and combination with free radicals to form a stable non-radical form (DPPH-H)^28,45^. The scavenging effect on DPPH radical of EULP-MAE was much higher than those of EULP-CHE at the concentrations ranged from 0.1 to 2.0 mg/mL, which was closely interrelated with the phenolic content of the extractum. At the sample concentration of 2 mg/mL, EULP-MAE exhibited pronounced DPPH radical scavenging activity of 84.3%, which was significantly higher than that of EULP-CHE (65.9%). The order of radical scavenging effect of EULP to DPPH in comparison with the control (commercial BHA and BHT) was BHA > EULP-MAE > EULP-CHE > BHT. In the present study, the increment of DPPH antioxidant effect of the polysaccharide fractions was in accordance with the increase of the average molecular weight. The IC50 values were 0.41, 0.87, 1.22 and 3.56 mg/mL for BHA, EULP-MAE, EULP-CHE, and BHT respectively. The results demonstrated that all the polysaccharide fractions had lower antioxidant capacity than BHA, but higher than BHT. In comparison with the high cost and the limited efficiency of artificial antioxidants, such as BHA and BHT, the extracted polysaccharides offered a relatively feasible source of natural antioxidant. Overall, the investigations of antioxidant effect *in vitro* suggested that the polysaccharides extracted by the MAE process demonstrated an improved antioxidant capacity and had promising prospects in potential applications as functional foods. It could improve aging and age-related diseases by reducing intracellular free radicals^46^. Owing to their safety and nontoxic properties, some of bioactive polysaccharides have also been widely used in biochemical and medical industries^47^.

## Conclusion

To improve the yield of polysaccharides from *E*. *ulmoides* leaf, the MAE technology was combined with RSM for the optimization of extraction process. On the basis of contour plots and variance analysis, the maximum polysaccharides yield of 12.31% was achieved by extraction at 74 °C for 15 min with a solid to liquid ratio of 1:29 g/mL, which was 2.9-fold higher than that of the CHE method and in good agreement with the predicted yield value (12.35%). Moreover, the extractum collected by the MAE process showed 1.7-fold higher yield of bioactive constituents as compared with the CHE process. The polysaccharides were identified to belong to the glucan family from the FTIR spectroscopy analysis. All these polysaccharides have similar compositions which were comprised of a large amount of glucose (38.2–39.1%) and a small quantity of other sugar components. The polysaccharides from optimized MAE process had a high sugar content (89.5%), together with a high molecular weight (*M*_w_ 38,830 g/mol) and polydispersity (*M*_w_/*M*_n_ 2.19), and exhibited high antioxidant activity (RSI 1.22). It was concluded that the MAE provided a promising alternative to isolate polysaccharides and bioactive components from *E*. *ulmoides* for potential application as natural antioxidants in food or biomedical industry.
